# Nutritional strategies for small ruminant gastrointestinal nematode management

**DOI:** 10.1093/af/vfae019

**Published:** 2024-10-14

**Authors:** Dan Quadros, Joan Burke

**Affiliations:** Department of Animal Science, University of Arkansas System Division of Agriculture, Little Rock, AR 72204, USA; U.S. Department of Agriculture/Agricultural Research Service, Dale Bumpers Small Farms Research Center, Booneville, AR 72927, USA

**Keywords:** diet, pasture, supplement, tannin, worm burden

**Implications**

- Pasture management can reduce the risk of gastrointestinal nematode infections in small ruminants and enhance the nutritive value of their diets.
- Supplementation, notably with protein, can increase the resistance and resilience of small ruminants to gastrointestinal nematodes.
- Copper oxide wire particles can reduce barber pole worm (*Haemonchus contortus*) infections in small ruminants and increase the efficacy of dewormers.
- Plants rich in condensed tannins can be an ally to sustainable integrated parasite management by controlling gastrointestinal nematodes and improving feed efficiency.

## Introduction

Gastrointestinal nematodes (GIN) are the most important health issue for small ruminants in many world regions. The GIN parasitism causes welfare concerns and severe economic losses related to reduced productivity, cost of treatment, and, eventually, mortality.

The most economically significant GIN in small ruminants belongs to the order Strongylida and the family Trichostrongylidae. They are found in different parts of the digestive tract according to the species. Barber pole worm (*Haemonchus contortus*) and brown stomach worm (*Teladorsagia circumcincta*), bankrupt worm (*Trichostrongylus colubriformis*), and nodular worm (*Oesophagostomum columbianum*), which are frequently associated with parasitic gastroenteritis, colonize the abomasum, small intestine, and large intestine, respectively.

**Figure 1.**
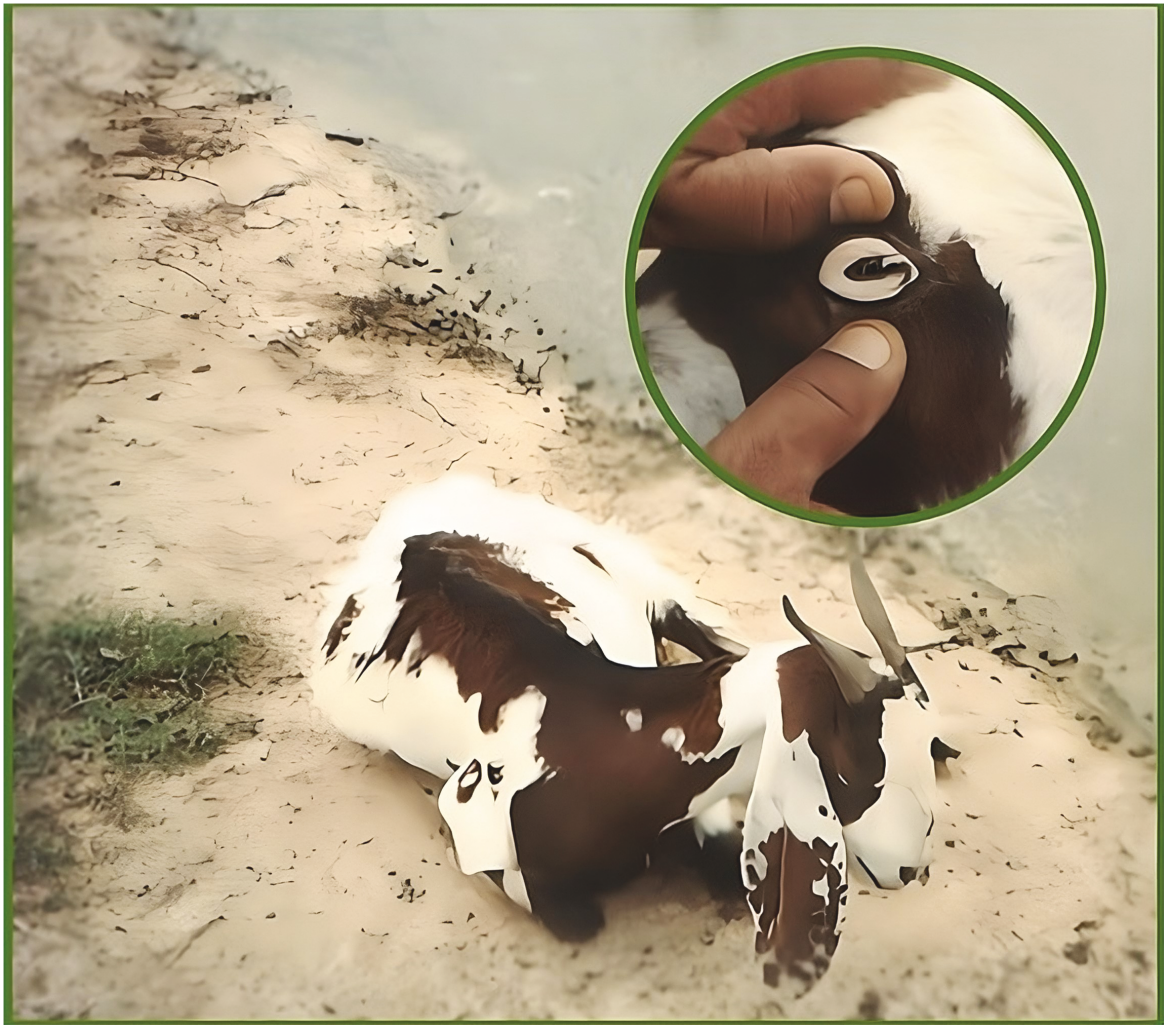
Goat with severe anemia caused by barber pole worm (*Haemonchus contortus*). The pale to white conjunctiva, weakness, lethargy, and sudden death are clinical signs of hemonchosis.

Barber pole worm is by far the most common and pathogenic GIN of small ruminants in tropical and subtropical regions due to the blood-feeding habit (chiefly linked to anemia; Figure 1) and high prolificacy, as a female can lay from 5,000 to 10,000 eggs per day.

Brown stomach worm is a major parasite species in temperate countries. Bankrupt worm invades the intestinal mucosa and causes lesions that develop as circular thickened areas several centimeters in diameter, while nodular worm larvae penetrate the large intestinal wall and become encysted, forming multifocal nodules throughout the lower gut.

**How do gastrointestinal nematodes infect small ruminants?** During the parasitic phase, adult worms live in the animals. Their eggs are shed in the animal’s feces, contaminating the pastures. The animal infection occurs via ingestion of larvae present in contaminated forage.

The control of GIN in small ruminants has mainly relied on using anthelmintics. With the failure of chemotherapy due to widespread parasite resistance to commercially available anthelmintics (Charlier et al., 2022) and concerns about drug residues in foods and ecotoxicological effects of drug excretion on the environment, researchers have studied other tools for integrated parasite management (IPM; Burke and Miller, 2020).

Feeding a balanced diet for maintenance, growth, and reproduction directly influences health since host resistance to infection is mediated predominantly through the involvement of its immune system (Atiba et al., 2020). Manipulating diets can be an essential tool to increase small ruminant resistance and resilience to GIN (Coop and Kyriazakis, 2001; Hoste et al., 2016).

**Resistance** is the ability to prevent or limit establishment or development of infection.

**Resilience** is the ability to maintain a reasonable production level when subjected to a parasitic challenge.

This article examines nutritional strategies as tools for small ruminant GIN management. First, we will uncover the impacts of GIN parasitism on nutrition and metabolism. Then, as most of the small ruminants are raised on pastures and get their nutrients from the forage plants, we will look at pasture management impacts on GIN parasitism and forage nutritive value. Following, we will describe how protein, energy, mineral, and vitamin supplementation can affect the host resistance and resilience. Next, we will discuss how the use of copper oxide wire particles (COWP), a dietary supplement, can control barber pole worm. Finally, we will explain how plant secondary compounds can be incorporated into small ruminant diets and contribute to IPM.

The different components of feed systems presented in this article are related to nutrition and interact with GIN management with the main goal to limit gastrointestinal parasitism in small ruminants by minimizing contamination of pastures and the contact of hosts with the infective third stage larvae, eliminating or disturbing the biology of GIN in the host, and improving host response (resistance and/or resilience; Torres-Acosta and Hoste, 2008).

## Impact of GIN on Nutrition and Metabolism

The primary pathophysiological effects of GIN on nutrition and metabolism that impair small ruminant production are reduction of voluntary feed intake, decreased feed efficiency, and changes in nutrient utilization (Hoste et al., 2016). A reduction of intake from 10% to 30% is commonly observed in subclinical infections (Coop and Sykes, 2002). Reduced feed intake directly impacts the level of nutrients ingested and, therefore, the animal response and function.

Loss of endogenous protein into the digestive tract due to leakage of plasma protein and increased mucoprotein can be substantial in parasitized small ruminants, exemplified by 10% loss of circulating blood per day in barber pole worm and 20 to 125 g protein per day in bankrupt worm infections in sheep (Coop and Kyriazakis, 2001). Blood losses can be much more significant than the blood consumption itself as barber pole worm uses the lancet to cut the abomasal tissue that induces hemorrhages. As a result, haemonchosis in sheep and goats can induce hematological alterations (hypoproteinemia, hypoalbuminemia, and hypoglobulinemia), anemia, and depletion of serum macro and trace minerals (e.g., P, Ca, Fe, Zn, and Cu; Coop and Sykes, 2002; Hoste et al., 2016; Atiba et al., 2020).

Parasitized animals not only have their digestion impaired due to the reduction of enzymatic functions and changes in digestive juice secretion but also suffer a decrease in nutrient absorption in the small intestine as a result of disrupted digestive process. Ultimately, nutrients are diverted from tissues (e.g., muscle, udder, wool follicles) to compensate and replace losses caused by GIN (Coop and Sykes, 2002; Athanasiadou et al., 2008).

**Figure 2.**
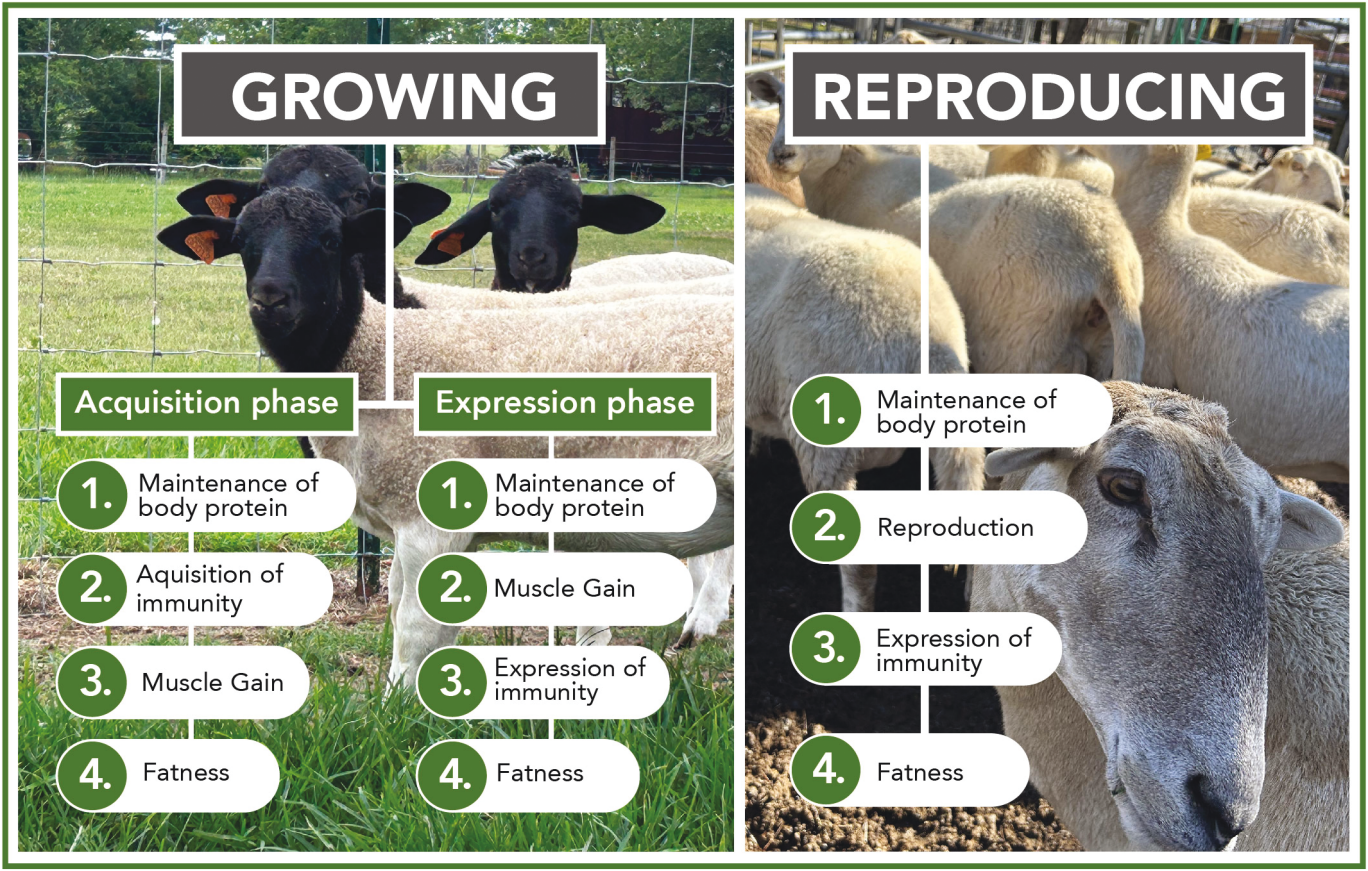
Possible ordering of priorities (1 highest to 4 lowest) given by a growing or a reproducing animal to its various body functions when partitioning a scarce food resource. For a naive, growing animal without any prior experience of a gastrointestinal nematode challenge, the phase of acquisition of immunity is considered separately from that of expression of immunity. Maintenance of body protein includes repair, replacement, and reaction to damaged or lost tissue. Source: Adapted from Coop and Kyriazakis (1999).

The allocation of absorbed nutrients varies according to the order of priority, depending on the physiological stage of the animals, such as growth and reproduction (Coop and Kyriazakis, 1999). In all physiological stages, maintenance of body protein, including repair, replacement, and reaction to damaged or lost tissue, is the number one priority (Figure 2).

As the allocation of nutrients to reproduction (pregnancy/lactation) is prioritized before the expression of acquired immunity, peri-parturient rise occurs, which is the rise in fecal parasitic output due to relaxation of acquired immunity to parasites around lambing/kidding (Coop and Sykes, 2002).

The depletion of intake, digestibility, and nutrient utilization, and consequently animal performance, depends on several factors related to both parasites (i.e., species and number, extent of larval challenge) and host (species, breed, age, physiological state, nutritional and immune status; Coop and Sykes, 2002; Athanasiadou et al., 2008; Hoste et al. 2016).

## Nutritional Strategies for Sustainable IPM

**Figure 3.**
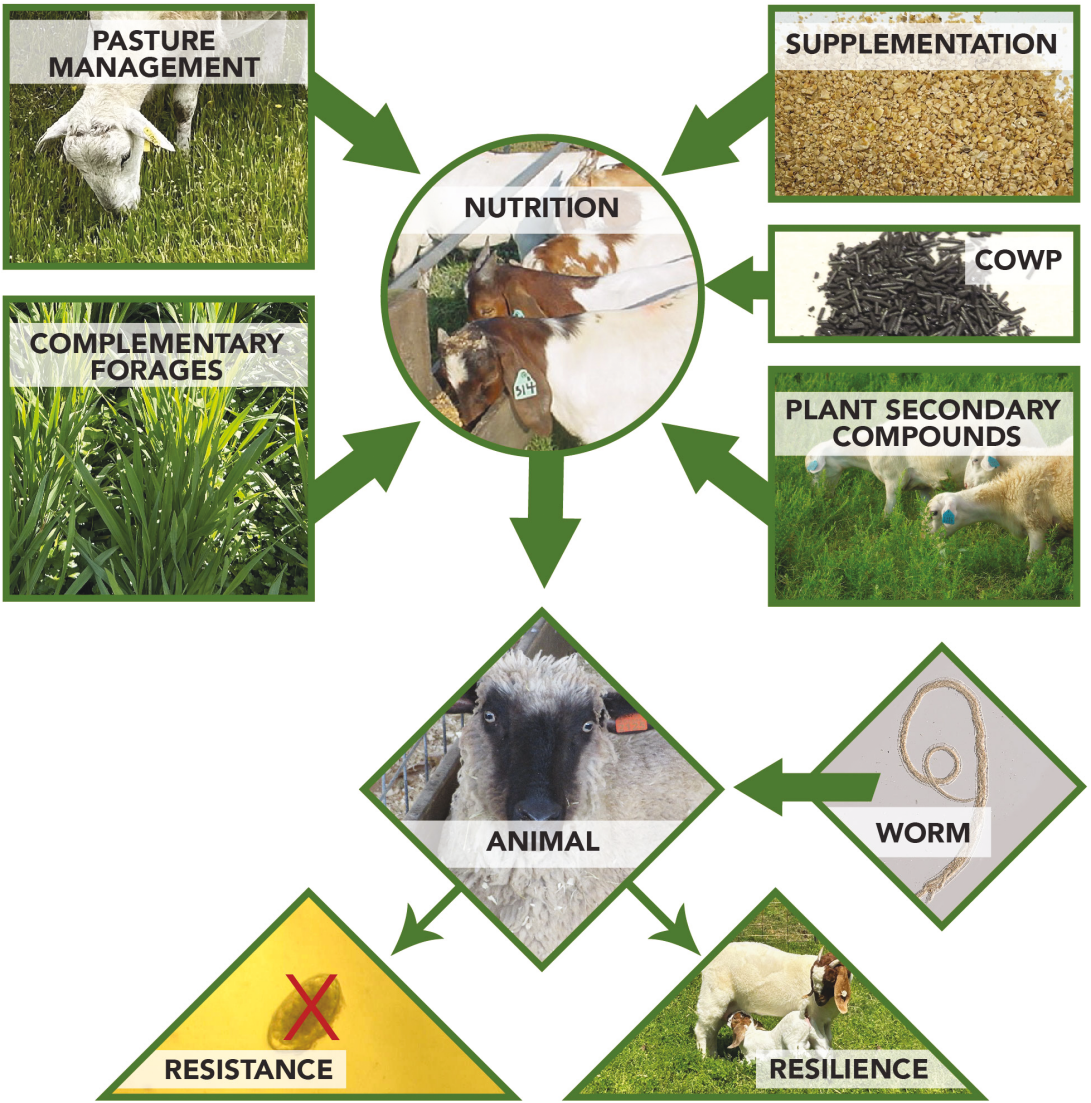
Nutritional strategies for small ruminant gastrointestinal nematode management. Pasture management (species, variety, environment, soil fertility, grazing system, defoliation frequency and intensity, and forage allowance), complementary forages (small grains, summer annuals, browsing, hay, silage, haylage, and agricultural byproducts), supplementation (protein, energy, minerals, and vitamins), copper oxide wire particles (COWP; dose, and frequency) and plant secondary compounds (present in legumes, herbs, shrubs, trees, and among others) affect nutrition. Nutrition (feed composition, digestibility, and intake) influences animal response (age, breed, natural resistance, production level, and physiological stage) to worms (species, number, and extension of the challenge), increasing resistance (by decreasing fecal egg counts and worm burden), and resilience (hematocrit and performance).

Nutrition strategies in a small ruminant IPM program should be seen in a holistic approach, in which its effects on host-parasite interaction are not isolated (Figure 3). First and foremost, pasture management and complementary forages can help to reduce the risk of GIN infections and provide good forage quality. Furthermore, pasture management can potentially increase soil organic matter and sequestrate carbon. Then, supplementation, which can reduce fecal egg counts (FEC; the gold standard to estimate worm burden in the live animal) and adult worm burdens, and increase performance. Among minerals, we highlight the use of COWP to control barber pole worm infections and increase the efficacy of dewormers. Finally, the inclusion of plant secondary compounds in sheep and goat diets (e.g., condensed tannins) as an ally to manage GIN, improve feed efficiency, and decrease methane emissions.

## Pasture Management to Reduce GIN Parasitism and Enhance Forage Nutritive Value

Grazing management is a crucial point in a sustainable IPM program because pastures are where most of the worms are located, in the form of eggs and larvae. We cannot dissociate the impacts of pasture management on larvae ingestion and forage nutritive value.

Rotational grazing allows producers to control stocking rate, how short plants are grazed, how long animals graze in a paddock, and grazing intervals in order to reduce the ingestion of infective larvae (Glennon, 2017; Burke and Miller, 2020; Bricarello et al., 2023). Rotational grazing also can provide forage with high nutritive value, impacting positively the host’s immune response. Co-grazing small ruminants with cattle and horses can reduce parasite transmission, as cross-infection usually has little significance (Bricarello et al., 2023).

Complementary forage plants also can contribute to a sustainable IPM. Incorporating annual forages into a pasture-based production system can reduce the risk of parasite infection in multiple ways. Warm- (e.g., pearl millet and Sudan grass) and cool-season (e.g., oats, rye, triticale, and wheat) annuals are known for their high nutritive value and can meet the energy and protein requirements of many classes of small ruminants. In addition to higher nutritive value compared to perennial grasses, planting these annuals will result in 45 to 60 days of plant growth before grazing, which reduces larvae availability (notably when there is some level of soil disturbance) and lowers the chance for the animals to ingest infective larvae when correctly managed (Glennon, 2017). Legumes and brassicas also are good options of complementary forages for small ruminants.

Allowing animals to browse on woodlot vegetation encourages them to eat higher in the canopy, where there is less chance of picking up parasites. Additionally, it can be used as a strategy to rest permanent pastures and provide an additional feed source while warm-season forages are growing. In silvopasture systems, which is the practice of integrating livestock, forage production, and forestry, it is possible to provide high-quality forage and plant secondary compounds while keeping animals from continuously grazing close to the ground (Glennon, 2017; Griffiths and Carr, 2022). This alternative is more suitable for goats, as they are browsers, than sheep, that are grazers. Because of inherent grazing behavior, goats seem to develop a lower immune response against GIN than sheep. In predominantly grass pastures, exposure to GIN larval challenge causes higher levels of infections in goats than sheep, differentially when the animals have the opportunity to browse (Hoste et al., 2008).

## Supplementation as a Tool to Increase Resistance and Resilience to GIN

Protein has been understandably the most studied nutrient in supplementation programs aiming to increase resistance and resilience of small ruminants to GIN due to its importance in repairing damaged tissues, offsetting endogenous protein losses, and its role in the host’s immune response (Coop and Sykes, 2002).

Protein supplementation can influence the different effector mechanisms of the immune response towards GIN such as proliferation of inflammatory cells (i.e., mucosal mast cells, globule leucocytes, eosinophils and goblet cells), associated release of highly proteinaceous effector molecules (e.g., mast cell proteases, leukotrienes, and mucoprotein-containing mucous), and production of immunoglobulins (e.g., IgA and IgE; Athanasiadou et al., 2008). Additionally, protein supplementation seems to improve host resilience by diverting metabolizable protein toward dealing with the detrimental effects of the infection and thus improving animal production and reproductive performances (Atiba et al., 2020).

**Figure 4.**
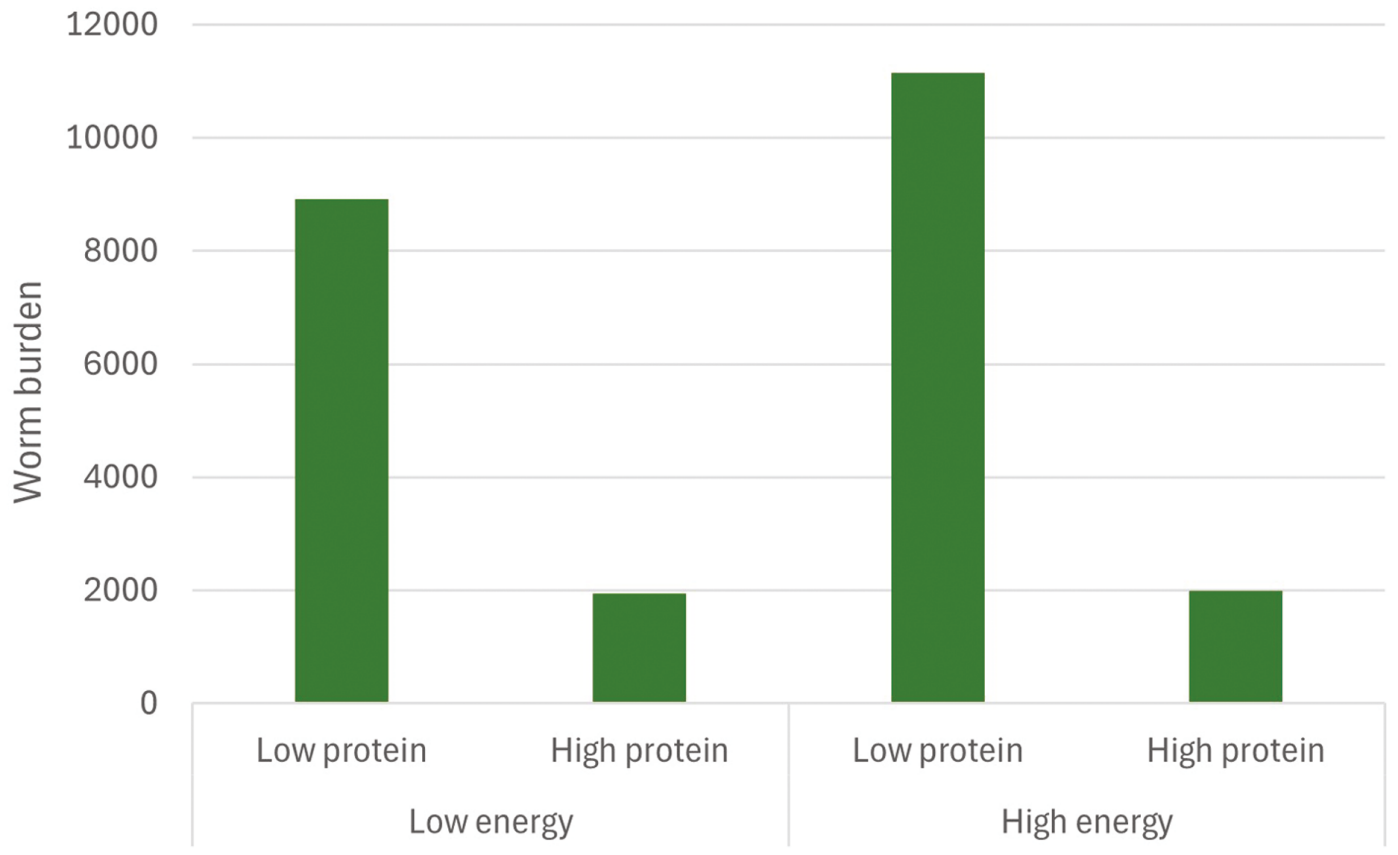
Average brown stomach worm (*Teladorsagia circumcincta*) burdens (geometric mean log_10_*x* + 1) of single- and twin-bearing ewes fed low- and high-protein and energy diets. Regardless of the level of dietary energy, increasing protein in sheep diets increased resistance to brown stomach worm by reducing 80% of worm burden. Source: Adapted from Donaldson et al. (1998).

High-protein diet (20% of crude protein) reduced brown stomach worm burdens in single- and twin-bearing ewes compared to low-protein diet (12% of crude protein), regardless of the level of dietary energy (low 70:30 vs. high 30:70, alfalfa hay:ground barley; Figure 4).

Increased protein in sheep diets during the early period of infection can influence the later stages of development of resistance and resilience to barber pole worm (Datta et al., 1999) and bankrupt worm (Kahn et al., 2000). Interestingly, feeding sheep a more proteaceous diet for a relatively short period (i.e., 9 weeks) can result in long-term benefits (i.e., over a year), probably due to enhanced immuno-responsiveness to GIN (Datta et al., 1999).

Supplementation can be used as a tool to boost the immune response in critical phases of small ruminant production, for instance, to reduce peri-parturient rise (Kahn et al. 2003) and post-weaning mortality (Coop and Kyriazakis, 2001).

The protein levels in relation to maintenance requirements and the demand for other physiological functions influence the potential for protein to enhance resistance to infection. However, the magnitude of improved host resistance following protein supplementation depends on the degree of protein scarcity in the absence of supplementation (Kahn et al., 2000).

Different protein feeds may reduce GIN infections and increase animal performance. For instance, fish meal supplementation can offset hypoproteinemia of predominantly barber pole worm parasitized sheep and significantly affect immunological responses indicated by FEC reductions and increased hematocrit and growth performance (Crawford et al., 2020). Supplementing sheep with soybean meal increased resilience, with fewer clinical signs of haemonchosis (i.e., anorexia, hypoproteinemia, hypoalbuminemia, weight loss, and edema), and resistance, with lower FEC and worm burdens (Wallace et al. 1995, 1996).

There is an interaction between nutrition and genetics on the host’s immune response, in which breeds or selected lines more resistant to GIN do not respond to protein supplements to overcome the adverse effects of parasitism as well as less resistant ones (Wallace et al. 1995, 1996; Khan et al. 2003).

The production level is another factor that affects the response of protein supplementation on resistance and/or resilience to GIN infections. Etter et al. (2000) found that low- and high-milk-producing goats infected with bankrupt worm had lower FEC when they received high protein diets (130% of the requirements), suggesting that resistance was enhanced by protein supplementation; however, only in high-producing does, the milk production and milk composition parameters were improved with high-protein diet.

Energy supplementation may also affect the resistance and resilience of small ruminants to GIN. Supplementing browsing kid goats, naturally infected with GIN (barber pole, bankrupt, and nodule worms) with 1.5% of the body weight (BW) of ground corn (covering 44% of energy and 35% of protein requirements) increased resistance (i.e., decreasing FEC, worm length, bankrupt worm burden and barber pole worm, and bankrupt worm fecundity) and resilience (i.e., increasing growth performance) of the animals compared to unsupplemented ones, bringing additional $5.8 USD per head (Gárate-Gallardo et al., 2015).

As supplementation is an accessible technology to be adopted by producers, the decision will be based on the parasitological challenge, animal’s genetics, forage availability and quality, and benefit:cost ratio. Grazing lambs majorly infected with barber pole worm (80%) fed with highly proteinaceous supplement (approximately 48% of crude protein), containing basically distillers’ grains with solubles (DDGS), corn gluten meal, soybean meal and fat, at a level of 1% BW, increased growth performance, FAMACHA^©^ scores and hematocrit while reduced FEC, compared with unsupplemented ones, mainly when lambs grazed predominantly tall fescue established pastures compared to newly sown pasture converted from cropland (Campbell et al., 2021). According to these authors, the improvement in lamb growth by 9.4 kg in 112 days could pay off supplementation costs and resulted in $1.27 USD per each $1.00 USD invested in supplementation.

Energy-protein supplementation (approximately 20% crude protein and 2.8 Mcal/kg metabolizable energy) at the level of 1.7% BW increased resistance (i.e., decreasing FEC, after the course of 69 days) and resilience (i.e., increasing growth performance and wool growth) of grazing lambs naturally infected with GIN, compared with unsupplemented ones (Tafernaberry et al., 2022). Using DDGS to replace approximately one-third of sorghum grain and half of soybean meal did not change the results when the supplement composition remained the same.

Harmonious functioning of the immune system requires the presence of several macro- and trace minerals. The concentrations of minerals required for healthy animals are often below what is required for animals experiencing an immunological challenge (Atiba et al., 2020). According to these authors, supplementation of copper, iron, and zinc can enhance host resistance against nematode infection, as shown by decreased FEC and worm burdens.

Lambs receiving mineral and vitamin supplementation decreased FEC and increased energy utilization and average daily gain, becoming a tool to be included in targeted selective treatment thresholds for controlling GIN in small ruminants (Hughes et al., 2023).

## COWP in an IPM Program

**Figure 5.**
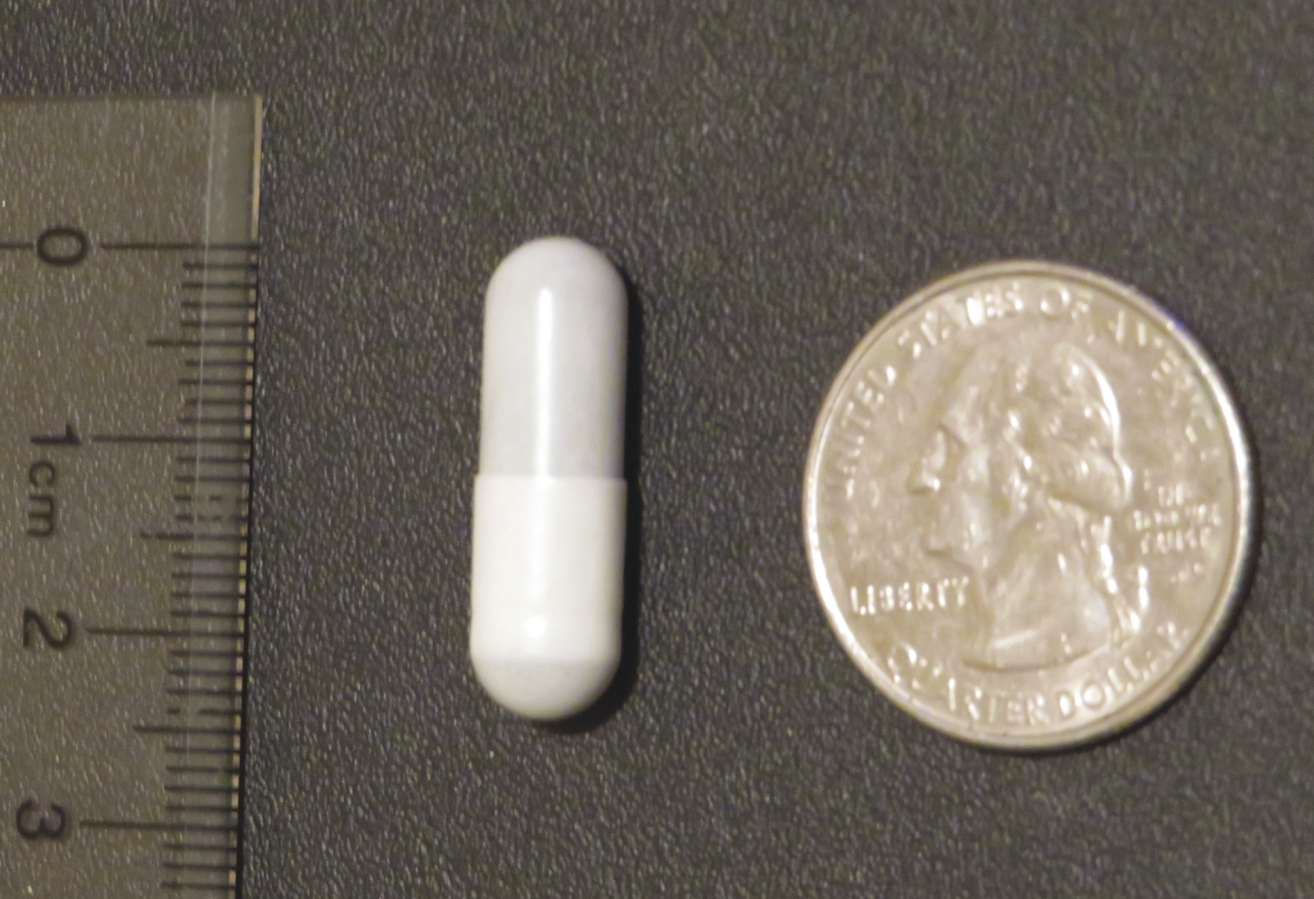
Copper oxide wire particles (COWP), 2 g capsule. COWP have anthelmintic activity against abomasal nematodes.

The COWP are a dietary supplement that became an alternative to anthelmintic drugs since they possess anthelmintic activity within 72 h after administration (Burke and Miller, 2020). The COWP are administered as a bolus (Figure 5) and are only effective against barber pole worm, not intestinal worms.

The COWP act on adult nematodes through the increased copper status of the host, or directly due to increased copper in the abomasum, which could potentially damage and penetrate the cuticle of barber pole worm (Burke and Miller, 2020).

The COWP are administered in low doses, inside gel capsules. The capsule is normally administered to the animal using a pill gun. From 0.5 to 1 g is recommended for lambs and kids, while for mature ewes and does the dose varies from 1 to 2 g, resulting in substantial FEC reduction within 7 days (Burke and Miller, 2020). A dose of 2 g in naturally infected sheep and goats reduced, respectively, FEC by 81% and 91%, and barber pole worm burden by 67% and 86%, 26 days after treatment (Soli et al., 2010).

Apparently, the anthelmintic activity of COWP does not last more than 72 hours, similar to the anthelmintic levamisole, but animals can be treated again after 4 to 6 weeks, if necessary, based on signs of anemia. There is no known parasite resistance to COWP, and its use increases the efficacy of the anthelmintics albendazole and levamisole (Burke and Miller, 2020). However, as sheep are very susceptible to copper toxicity, which can result in death, is recommended that copper levels be monitored, and the lowest dose used.

The effectiveness of COWP may be greater in combination with a high-nutrition plan and feeding or grazing sericea lespedeza, a forage rich in condensed tannins (Burke and Miller, 2020).

## Plant Secondary Compounds to Reduce Parasite Infections and Improve Feed Efficiency

The plant secondary compounds with anthelmintic activity include tannins, lactones, alkaloids, saponins, terpenes, glycosides, and phenolic compounds. Some of them, depending on the amount ingested, can reduce feed intake, induce nutritional deficiencies, and trigger neurological effects (Burke and Miller, 2020). However, when used in moderation or when restricted to high-risk exposure to GIN or even coccidia, plant secondary compounds have the potential for controlling GIN in small ruminants (Hoste et al., 2015; Lima et al., 2019). The category of these compounds that has received the most attention regarding anti-helminthic properties is condensed tannins (Griffiths and Carr, 2022).

Plants rich in condensed tannins with demonstrated activity against GIN in sheep and goats include sericea lespedeza (*Lespedeza cuneata*), sainfoin (*Onobrychis viciifolia*), sulla (*Hedysarum coronarium*), black wattle (*Acacia mearnsii)*, big trefoil (*Lotus pedunculatus*), birdsfoot trefoil (*Lotus corniculatus*), among others (Hoste et al., 2015, 2016; Lima et al., 2019; Terrill, 2024).

Condensed tannins can have direct and indirect effects on GIN. The direct anthelmintic effects are associated with the binding of larval proteins, which slows egg hatching, larval development, and exsheathment, while indirect effects are associated with improvements to protein metabolism and immune function within the animal (Hoste et al., 2015; Correa et al., 2020; Griffiths and Carr, 2022).

Goats and sheep fed sericea lespedeza (grazing, hay, leaf meal, pellets, and silage) decreased FEC, larval development, and worm burdens, showing potential to be used in an IPM, particularly when dealing with barber pole worm (Terrill, 2024). However, prolonged feeding (i.e., past 6 weeks) can lead to the binding of some trace minerals, reducing their availability to the animal.

Besides the anthelmintic effects, condensed tannins alter the fermentation products in the rumen, such as volatile fatty acids proportions and methane, which can increase feed efficiency (Correa et al., 2020; Pech-Cervantes et al., 2021). Methane, although necessary to remove the metabolic hydrogen in the rumen, represents energy loss in the digestion process and it is an environmental concern as the most critical greenhouse gas related to ruminant production.

## Conclusion

Nutrition is an essential tool in a sustainable IPM program for small ruminants, helping to increase resistance and resilience when animals face GIN challenges.

Pasture management and the use of complementary forages; protein, energy, minerals, and vitamins supplementation; utilization of COWP; and the inclusion of plant with secondary compounds (e.g., condensed tannins) in the diets are nutritional strategies that producers should adopt to overcome gastrointestinal infections in sheep and goats.

Future research should consider how to incorporate the different nutritional strategies in feeding systems and evaluate their effects on animal health, more specifically GIN infections, animal performance, and efficiency and sustainability of small ruminant production.

## About the Authors

 **Dan Quadros** is an assistant professor and small ruminant specialist at the University of Arkansas System Division of Agriculture (UADA). He received his PhD and master’s degree in animal science from Sao Paulo State University in Brazil, a second master’s degree in sustainability systems from the Pennsylvania State University in the USA, and a bachelor’s degree in agronomy from Southwest Bahia State University in Brazil. Additionally, he has post-doctoral training at the University of Florida and Texas A&M University, United States. His program at UADA is designed to discover solutions and deliver science-based information to develop sheep and goats value chains, using multiple extension methods to increase producer knowledge and understanding of small ruminant husbandry/health, production, and marketing. He is a member of the American Consortium for Small Ruminant Parasite Control. **Corresponding author:**dquadros@uada.edu

 **Joan Burke** is a research animal scientist in the U.S. Department of Agriculture (USDA)–Agricultural Research Service (ARS) Dale Bumpers Small Farms Research Center. She received a Ph.D. in reproductive biology at Oregon State University, a master’s in animal science from the University of Maine, and a bachelor’s degree from Cornell University, in the United States. Dr. Burke has been with ARS since 1999 where she has conducted research on the control of gastrointestinal nematodes in sheep and goats. Her program focuses on organic and grass-based production systems for small ruminant producers and includes development of alternatives to synthetic anthelmintics such as specialty forages, genetic and genomic selection for parasite resistance, nutrition and copper oxide wire particles. She is one of the founding members of the American Consortium for Small Ruminant Parasite Control.

## References

[CIT0001] Athanasiadou, S., J. Houdijk, and I. Kyriazakis. 2008. Exploiting synergisms and interactions in the nutritional approaches to parasite control in sheep production systems. Small Rumin. Res. 76(1-2):2–11. doi:10.1016/j.smallrumres.2007.12.016

[CIT0002] Atiba, E.M., S. Zewei, and Z. Qingzhen. 2020. Influence of metabolizable protein and minerals supplementation on detrimental effects of endoparasitic nematodes infection in small ruminants. Trop. Anim. Health Prod. 52(5):2213–2219. doi:10.1007/s11250-020-02275-w 32388661

[CIT0003] Bricarello, P.A., C. Longo, R.A. Rocha, and M.J. Hötzel. 2023. Understanding animal-plant-parasite interactions to improve the management of gastrointestinal nematodes in grazing ruminants. Pathogens. 12:531. doi:10.3390/pathogens12040531 37111417 PMC10145647

[CIT0004] Burke, J.M., and J.E. Miller. 2020. Sustainable approaches to parasite control in ruminant livestock. Vet. Clin. North Am. Food Anim. Pract. 36(1):89–107. doi:10.1016/j.cvfa.2019.11.007 32029191

[CIT0005] Campbell, B.J., A.E. Marsh, E.M. Parker, J.S. McCutcheon, F.L. Fluharty, and A.J. Parker. 2021. The effects of protein supplementation and pasture maintenance on the growth, parasite burden, and economic return of pasture-raised lambs. Transl. Anim. Sci. 5(3):txab113. doi:10.1093/tas/txab113 34316541 PMC8309954

[CIT0006] Charlier, J., D.J. Bartley, S. Sotiraki, M. Martinez-Valladares, E. Claerebout, G. von Samson-Himmelstjerna, S.M. Thamsborg, H. Hoste, E.R. Morgan, and L. Rinaldi. 2022. Anthelmintic resistance in ruminants: challenges and solutions. Adv. Parasitol. 115:171–227. doi:10.1016/bs.apar.2021.12.002 35249662

[CIT0008] Coop, R.L. and, I. Kyriazakis. 2001. Influence of host nutrition on the development and consequences of ne-matode parasitism in ruminants. Trends Parasitol. 17:325–330. doi:10.1016/s1471-4922(01)01900-6 11423375

[CIT0007] Coop, R.L., and I. Kyriazakis. 1999. Nutrition–parasite interaction. Vet. Parasitol. 84(3-4):187–204. doi:10.1016/s0304-4017(99)00070-9 10456415

[CIT0009] Coop, R. L., and A. R. Sykes. 2002. Interactions between gastrointestinal parasites and nutrients. In: M. Freer, and H. Dove, editors. Sheep nutrition. Wallingford, UK: CAB International; p. 313–331.

[CIT0010] Correa, P.S., L.W. Mendes, L.N. Lemos, P. Crouzoulon, V. Niderkorn, H. Hoste, L.M. Costa-Júnior, S.M. Tsai, A.P. Faciola, A.L. Abdalla, et al. 2020. Tannin supplementation modulates the composition and function of ruminal microbiome in lambs infected with gastrointestinal nematodes. FEMS Microbiol. Ecol. 96(3):fiaa024. doi:10.1093/femsec/fiaa024 32053145

[CIT0011] Crawford, C.D., D.J. Mata-Padrino, D.P. Belesky, and S.A. Bowdridge. 2020. Effects of supplementation containing rumen by-pass protein on parasitism in grazing lambs. Small Rumin. Res. 190:106161. doi:10.1016/j.smallrumres.2020.106161

[CIT0012] Datta, F.U., J.V. Nolan, J.B. Rowe, G.D. Gray, and B.J. Crook. 1999. Long-term effects of short-term provision of protein-enriched diets on resistance to nematode infection, and live-weight gain and wool growth in sheep. Int. J. Parasitol. 29(3):479–488. doi:10.1016/s0020-7519(98)00209-4 10333332

[CIT0013] Donaldson, J., M.F.J. Van Houtert, and A.R. Sykes. 1998. The effect of nutrition on the periparturient parasite status of mature ewes. Anim. Sci. 67(3):523–533. doi:10.1017/s1357729800032951

[CIT0014] Etter, E., H. Hoste, C. Chartier, I. Pors, C. Koch, C. Broqua, and H. Coutineau. 2000. The effect of two levels of dietary protein on resistance and resilience of dairy goats experimentally infected with *Trichostrongylus colubriformis*: comparison between high and low producers. Vet. Res. 31(2):247–258. doi:10.1051/vetres:2000120 10779203

[CIT0015] Gárate-Gallardo, L., J.F. J. Torres-Acosta, A.J. Aguilar-Caballero, C.A. Sandoval-Castro, R. Cámara-Sarmiento H.L. Canul-Ku. 2015. Comparing different maize supplementation strategies to improve resilience and resistance against gastrointestinal nematode infections in browsing goats. Parasite. 22:19. doi:10.1051/parasite/2015019 26071051 PMC4464326

[CIT0017] Glennon, H. (2017) Pasture management. American consortium for small ruminant parasite control. fact sheet. – [Accessed April 10, 2024]. Available from https://www.wormx.info/_files/ugd/6ef604_dae47e49103341dbb260f69318397cd3.pdf?fbclid=IwZXh0bgNhZW0CMTAAAR15v0LP2cMWKaOZaPjnnT8qy3-0Sr6bfIoiB3v9_fv4xumcRute7w96m-I_aem_Ac3dhzAss0Qnq6rt8ADCirTrrhIM7yxw_Fsrz_dbuok0W8NLApRQi4uuDb4IH2AsGQUBu14yxnWrtuX5PLpQOjaF.

[CIT0016] Griffiths, H., and A.N. Carr. 2022. Potential for silvopastoral systems to control nematode burden in livestock farming in winter rainfall areas of South Australia, Australia. Int. J. Vet. Sci. Res. 8:118–126. doi:10.17352/ijvsr

[CIT0018] Hoste, H., J.F. J. Torres‐Acosta A.J. Aguilar‐Caballero. 2008. Nutrition–parasite interactions in goats: is immunoregulation involved in the control of gastrointestinal nematodes? Parasite Immunol. 30:79–88. doi:10.1111/j.1365-3024.2007.00987.x 18186768

[CIT0020] Hoste, H., J.F. J. Torres-Acosta, J. Quijada, I. Chan-Perez, M.M. Dakheel, D.S. Kommuru, I. Mueller-Harvey and T.H. Terrill. 2016. Interactions between nutrition and infections with *Haemonchus contortus* and related gastrointestinal nematodes in small ruminants. Adv. Parasitol. 93:239–351. doi:10.1016/bs.apar.2016.02.025 27238007

[CIT0019] Hoste, H., J.F. J. Torres-Acosta, C.A. Sandoval-Castro, I. Mueller-Harvey, S. Sotiraki, H. Louvandini, S.M. Thamsborg T.H. Terrill. 2015. Tannin containing legumes as a model for nutraceuticals against digestive parasites in livestock. Vet. Parasitol. 212:5–17. doi:10.1016/j.vetpar.2015.06.026 26190131

[CIT0022] Kahn, L.P., M.R. Knox, G.D. Gray, J.M. Lea, and S.W. Walkden-Brown. 2003. Enhancing immunity to nematode parasites in single-bearing Merino ewes through nutrition and genetic selection. Vet. Parasitol. 112(3):211–225. doi:10.1016/s0304-4017(02)00438-7 12591197

[CIT0031] Hughes, M., E.J. Phillips, and R.A. Jones. 2023. Supplementation of minerals and vitamins influences optimal targeted selective treatment thresholds for the control of gastro-intestinal nematodes in lambs. Vet. Parasitol. 322:110026. doi:10.1016/j.vetpar.2023.110026 37713957 10.1016/j.vetpar.2023.110026

[CIT0021] Kahn, L.P., I. Kyriazakis, F. Jackson, and R.L. Coop. 2000. Temporal effects of protein nutrition on the growth and immunity of lambs infected with *Trichostrongylus colubriformis*. Int. J. Parasitol. 30(2):193–205. doi:10.1016/s0020-7519(99)00192-7 10704602

[CIT0023] Lima, P.D.M.T., P. Crouzoulon, T.P. Sanches, G. Zabré, A. Kabore, V. Niderkorn, H. Hoste, A.F.T. Amarante, L.M. Costa-Júnior, A.L. Abdalla, et al. 2019. Effects of *Acacia mearnsii* supplementation on nutrition, parasitological, blood parameters and methane emissions in Santa Inês sheep infected with *Trichostrongylus colubriformis* and *Haemonchus contortus*. Exp. Parasitol. 207:107777. doi:10.1016/j.exppara.2019.107777 31626795

[CIT0024] Pech-Cervantes, A.A., T.H. Terrill, I.M. Ogunade, and Z.M. Estrada-Reyes. 2021. Meta-analysis of the effects of dietary inclusion of sericea lespedeza (*Lespedeza cuneata*) forage on performance, digestibility, and rumen fermentation of small ruminants. Livest Sci. 253:104707. doi:10.1016/j.livsci.2021.104707

[CIT0025] Soli, F., T.H. Terrill, S.A. Shaik, W.R. Getz, J.E. Miller, M. Vanguru, and J.M. Burke. 2010. Efficacy of copper oxide wire particles against gastrointestinal nematodes in sheep and goats. Vet. Parasitol. 168(1-2):93–96. doi:10.1016/j.vetpar.2009.10.004 19931291

[CIT0026] Tafernaberry, A., E. Romaniuk, E.V. Lier, R. Reyno, and I. De Barbieri. 2022. High performance of growing lambs grazing *Paspalum notatum* INIA Sepé with energy-protein supplement including sorghum-DDGS. Agrocienc. Urug. 26:549. doi:10.31285/AGRO.26.549

[CIT0027] Terrill, T. 2024. American consortium for small ruminant parasite control investigations on the use of plant secondary compounds of sericea lespedeza for the control of sheep and goat parasites. Trop. Subtrop. Agroecosyst. 27(1):003. doi:10.56369/tsaes.4542

[CIT0028] Torres-Acosta, J.F.J., and H. Hoste. 2008. Alternative or improved methods to limit gastro-intestinal parasitism in grazing sheep and goats. Small Rumin, Res. 77(2-3):159–173. doi:10.1016/j.smallrumres.2008.03.009

[CIT0029] Wallace, D.S., K. Bairden, J.L. Duncan, G. Fishwick, M. Gill, P.H. Holmes, Q.A. McKellar, M. Murray, J.J. Parkins, and M.J. Steer. 1995. Influence of supplementation with dietary soyabean meal on resistance to haemonchosis in Hampshire down lambs. Res. Vet. Sci. 58:232–237. doi:10.1016/0034-5288(95)90108-6 7659847

[CIT0030] Wallace, D.S., K. Bairden, J.L. Duncan, G. Fishwick, M. Gill, P.H. Holmes, Q.A. McKellar, M. Murray, J.J. Parkins, and M.J. Steer. 1996. Influence of soyabean meal supplementation on the resistance of Scottish blackface lambs to haemonchosis. Res. Vet. Sci. 58:138–143. doi:10.1016/s0034-5288(96)90008-9 8685535

